# Obinutuzumab exposure during early twin pregnancy in antisynthetase syndrome with interstitial lung disease

**DOI:** 10.1016/j.ero.2026.100221

**Published:** 2026-08-05

**Authors:** Sarah Dyball, Ellen Bruce, Kim MacLeod, Ben Parker

**Affiliations:** Kellgren Centre for Rheumatology, Manchester Foundation Trust, Manchester, UK; Centre for Musculoskeletal Research, Faculty of Biology, Medicine and Health, The University of Manchester, Manchester, UK; Kellgren Centre for Rheumatology, Manchester Foundation Trust, Manchester, UK; St Mary’s Hospital, Manchester Foundation Trust,Manchester, UK; Kellgren Centre for Rheumatology, Manchester Foundation Trust, Manchester, UK; NIHR Manchester Biomedical Research Centre, Manchester University Hospitals NHS Foundation Trust, Manchester Academic Health Science Centre, Manchester, UK

To the Editor,

Antisynthetase syndrome (anti–Jo-1 positive) with inflammatory myopathy and interstitial lung disease (ILD) poses substantial challenges in pregnancy, particularly in patients with severe, treatment-refractory disease[1]. We report the first case of obinutuzumab administered during early pregnancy in a high-risk twin gestation, resulting in sustained maternal disease control and reassuring short-term neonatal outcomes.

The patient was diagnosed in 2015 with antisynthetase syndrome complicated by severe ILD, recurrent myositis flares requiring hospitalisation, and prior myocarditis. Her previous pregnancy in 2019 was complicated by placental failure, intrauterine growth restriction, and early delivery at 36 weeks. Standard immunosuppression was ineffective or poorly tolerated, and rituximab-induced anaphylaxis precluded further use. Obinutuzumab (1 g, days 1 and 14) in 2022 induced sustained remission with improved pulmonary function (forced vital capacity [FVC] from 53% to 75% predicted; diffusing capacity of the lungs for carbon monoxide [DLCO] from 36% to 52% predicted).

During preconception counselling, a single 1 g dose of obinutuzumab was administered in December 2023, with conception planned 2 to 3 months later to minimise foetal exposure. The patient subsequently did not attend follow-up and received no maintenance immunosuppression.

She re-presented in August 2025 at 6 weeks’ gestation after conceiving spontaneously with a dichorionic diamniotic twin pregnancy and was receiving no immunosuppressive therapy. The decision to administer obinutuzumab was made after multidisciplinary review, acknowledging the uncertainty of foetal exposure risk and substantial risk of maternal disease flare through pregnancy. In the event of a flare, the combination of previous poor response to standard therapy, presence of significant lung and cardiac disease, and a twin pregnancy posed a very high risk of adverse maternal and foetal outcomes.

Obinutuzumab 1 g was administered at 7 + 3 and 9 + 3 weeks’ gestation. Obinutuzumab is a humanised immunoglobulin (Ig)G1 monoclonal antibody with a terminal half-life of approximately 28 days[2]. Although obinutuzumab was administered in the first trimester, persistence of maternal circulating drug, together with increasing FcRn-mediated placental IgG transfer from the second trimester onwards, may have resulted in foetal exposure after the emergence of CD20-positive foetal B cells[3].

Maternal disease remained quiescent during pregnancy, with stable pulmonary function. Peripheral CD19⁺ B cells remained undetectable at 30 weeks’ gestation. Pregnancy was complicated by foetal growth restriction in 1 twin, and delivery was by elective caesarean section at 32 + 4 weeks. Table 1 summarises the maternal, obstetric and neonatal outcomes following antenatal obinutuzumab exposure. Both neonates required neonatal intensive care unit (NICU) care for prematurity. Immune evaluation showed low, but measurable, CD19-positive B-cell counts in both twins, 19 and 3 cells/µL, respectively. IgG and neutrophil counts were within local age-specific reference intervals, whereas IgM and total white cell counts were below the local reference range. No early serious or opportunistic infections occurred. The patient flared 2 weeks postpartum and was managed with glucocorticoids and further obinutuzumab with clinical improvement.

**Table 1:** Perinatal outcomes and early neonatal immune parameters following first trimester obinutuzumab exposure in a twin pregnancy

	Twin 1	Twin 2	Reference
Sex	Male	Female	
Gestation at delivery	32 + 4	32 + 4	
Birth weight	1342 g	1538 g	
Birth weight centile	Between 2nd and 9th centile	Between 9th and 25th centile	
Apgar score at 1 min	8	7	
Apgar score at 5 min	10	9	
Cord pH	7.17	7.27	
NICU admission	Yes	Yes	
Duration of NICU stay (d)	29	25	
CD19 positive B cells	19	3	40-1100 cells/µL
IgG	9.93	9.98	5.00-17.00 g/L
IgM	<0.05	<0.05	0.05-0.20 g/L
Total WCC	4.7	4.8	5.00-20.00 × 10⁹/L
Neutrophils	1.64	1.80	1.00-9.00 × 10⁹/L
Lymphocytes	2.30	2.42	2.00-10.00 × 10⁹/L
Early infection outcomes	No infections	No infections	

Reference ranges for neonatal haematology, immunoglobulins, and lymphocyte subsets were taken from local laboratory age-specific reference intervals. Birthweight centile was calculated using the RCPCH UK-WHO Neonatal and Infant Close Monitoring growth chart, adjusted for gestational age and infant sex. Early infection was defined as clinically suspected or microbiologically confirmed infection requiring antimicrobial treatment within the first 28 days of life. CD19, cluster of differentiation 19; IgG, immunoglobulin G; IgM, immunoglobulin M; NICU, neonatal intensive care unit; RCPCH, Royal College of Paediatrics and Child Health; UK WHO, United Kingdom World Health Organization; WCC, white cell count.

Initially, it was advised that breastfeeding could be considered, given the timing of obinutuzumab exposure in early pregnancy and the expected decline in circulating drug levels by delivery. However, following postpartum disease flare and further obinutuzumab treatment 5 weeks postpartum, she was advised not to breastfeed. This was discussed as part of the consent process for retreatment.

The infants received paediatric immunology input. Rotavirus vaccination was advised against because this is a live vaccine and both infants had low CD19 positive B-cell counts. Routine nonlive vaccinations were advised. Paediatric immunology follow-up was arranged at 6 months to reassess immune recovery and guide subsequent vaccination, including measurement of vaccine responses where appropriate.

This case describes first-trimester obinutuzumab exposure in a twin pregnancy complicated by severe antisynthetase syndrome with ILD. Maternal disease remained controlled during pregnancy, and short-term neonatal outcomes were reassuring, although neonatal B-cell lymphopenia was observed. Data are limited; further reporting of pregnancy exposures and longer-term infant follow-up are needed, and careful multidisciplinary counselling remains essential.

## CRediT authorship contribution statement

**Sarah Dyball:** Writing – review & editing, Writing – original draft, Conceptualization. **Ellen Bruce:** Writing – review & editing, Conceptualization. **Kim MacLeod:** Writing – review & editing, Conceptualization. **Ben Parker:** Writing – review & editing, Writing – original draft, Conceptualization.
